# The Contribution of Geriatric Educators to Public Policy

**DOI:** 10.1093/geroni/igaa057.1800

**Published:** 2020-12-16

**Authors:** Anna Faul, Jennifer Severence, Leland Waters

**Affiliations:** 1 University of Louisville Trager Institute, Louisville, Kentucky, United States; 2 University of North Texas, Fort Worth, Texas, United States; 3 Virginia Commonwealth University, Richmond, Virginia, United States

## Abstract

Despite the current pressure to reduce state and federal spending, policymakers must find ways to address the challenges of a growing population of older adults with complex health care problems. There is an increased need for the health professions workforce to have collaborative care skills and geriatric clinical competencies. Therefore, programs like the Geriatric Workforce Enhancement Program (GWEP) and the Geriatric Academic Career Awards (GACA) are important in strengthening the workforce and supporting policy development that addresses increased demands on the health care system. In 2019, the Bureau of Health Professions, under the Health Resources and Services Administration, provided 48 GWEP awards and 26 GACA awards in 37 states and 2 territories. These programs play an important advocacy role to improve on and expand geriatric education. This symposium provides an overview of these programs and their role in advancing geriatric care and in shaping policy.

